# Circulating Extracellular Vesicles Downregulate *NOS3* Expression in Endothelial Cells in Atrial Fibrillation

**DOI:** 10.3390/jcm15041399

**Published:** 2026-02-10

**Authors:** Nyozin Leimon, Anna Suzuki, Kohei Kawajiri, Giichi Nitta, Junji Yamaguchi, Satoshi Iwamiya, Satomi Hamada, Yasuhiro Shirai, Lai Wei, Masahiro Yamazoe, Kensuke Ihara, Tetsushi Furukawa, Tetsuo Sasano

**Affiliations:** 1 Department of Cardiovascular Medicine, Institute of Science Tokyo, Tokyo 113-8519, Japan; nyozinleimon.cvm@tmd.ac.jp (N.L.); kohei-kawajiri.cvm@tmd.ac.jp (K.K.); giichi-nitta.cvm@tmd.ac.jp (G.N.); yamaguchi.j.b1a4@m.isct.ac.jp (J.Y.); satoshi-iwamiya.cvm@tmd.ac.jp (S.I.); hamamlab@tmd.ac.jp (S.H.); weilcvm@tmd.ac.jp (L.W.); yamazoe.masahiro@tmd.ac.jp (M.Y.); iharcvm@tmd.ac.jp (K.I.); 2 Department of Clinical Laboratory, Institute of Science Tokyo Hospital, Tokyo 113-8519, Japan; 3 Department of Cardiology, AOI Universal Hospital, Kawasaki 210-0822, Japan; whity_yasuo@yahoo.co.jp; 4 Institute of Science Tokyo, Tokyo 113-8519, Japan; t_furukawa.bip@mri.tmd.ac.jp

**Keywords:** atrial fibrillation, endothelial dysfunction, extracellular vesicles, *NOS3*

## Abstract

**Background:** Atrial fibrillation (AF) is closely linked to endothelial dysfunction, yet its mechanisms remain unclear. Extracellular vesicles (EVs), including exosomes, are released by most cell types and mediate intercellular communication. We therefore investigated the role of EVs in endothelial dysfunction associated with AF. **Methods:** Vascular endothelial function in patients with sinus rhythm (SR), premature ventricular contractions (PVCs), or AF was assessed by peripheral arterial tonometry. Plasma-derived EVs were isolated from these three groups. Conditioned medium was collected from cultured cardiomyocytes (CMs), which were paced either regularly or irregularly at 1 Hz or 10 Hz or were non-paced, and EVs were subsequently isolated from the conditioned media. The isolated EVs were applied to endothelial cells (ECs), and mRNA levels of vasoactive genes were quantified. **Results:** The reactive hyperemia index (RHI) was significantly lower in patients with AF than in those with SR (RHI values: 1.98 in SR vs. 1.57 in AF, *p* = 0.049), whereas no significant decrease was observed in patients with PVCs, indicating endothelial dysfunction in AF. The plasma EV concentration was significantly higher in patients with AF than in those with SR. CMs subjected to 10 Hz irregular pacing released more EVs than non-paced cells and cells under 1 Hz regular pacing. When applied to ECs, EVs from patients with AF and from rapidly paced CMs significantly reduced *NOS3* mRNA expression in vitro. **Conclusions:** Circulating EVs are increased in AF and could be associated with an impaired endothelial function in AF.

## 1. Introduction

Atrial fibrillation (AF) is the most common sustained cardiac arrhythmia in clinical practice and causes an irregular peripheral arterial pulse and flow. During AF, disorganized and rapid atrial electrical activity abolishes effective atrial contraction, which promotes blood stasis, particularly within the left atrial appendage, and predisposes to thrombus formation. Such thrombi can embolize, causing ischemic stroke and other systemic embolic events [1].

Endothelial cells (ECs) form a single-cell monolayer lining the inner surface of the blood vessels. The endothelium plays diverse physiological roles and is critically involved in vascular pathology [2]. In particular, impaired nitric oxide (NO) bioavailability in ECs markedly compromises endothelium-dependent vasodilation. Endothelial NO production is largely mediated by endothelial NO synthase (eNOS), which is encoded by the *NOS3* gene; thus, reduced eNOS expression is closely linked to decreased NO production, resulting in endothelial dysfunction [3,4,5,6]. Endothelial dysfunction is a key contributor to the development and progression of cardiovascular disease, and non-invasive assessments such as flow-mediated dilation and reactive hyperemia peripheral arterial tonometry provide prognostic information for future cardiovascular events [7]. The reactive hyperemia index (RHI) is considered a useful surrogate of endothelial function—particularly vasodilatory capacity—and is thought to reflect NO-dependent vasodilation [8,9].

Studies have shown that endothelial dysfunction is closely associated with AF. In patients with AF, reduced eNOS expression and consequent endothelial dysfunction have been reported [10]. Polovina M, et al. further demonstrated that sustained AF is associated with endothelial dysfunction even in young patients without structural heart disease or conventional cardiovascular risk factors [11]. Notably, an impaired vascular endothelial function has been reported to improve after restoration of the sinus rhythm by electrical cardioversion or catheter ablation, suggesting that AF itself may contribute to endothelial dysfunction [12,13]. Although AF-related irregular pulse patterns and altered hemodynamics have been proposed as potential contributors to reduced eNOS expression, the mechanisms linking AF to eNOS downregulation and endothelial dysfunction have not yet been fully elucidated [14].

Nano-sized membrane-bound extracellular vesicles (EVs), including exosomes, are released by most cell types and can carry diverse bioactive cargo such as nucleic acids, lipids, proteins, and metabolites [15]. EVs participate in a wide range of physiological and pathological processes and serve as important mediators of intercellular communication [16]. Importantly, EV function is altered not only by the type of the secreting cell but also by its physiological state. de Jong et al. reported an alteration in the RNA and protein composition of the EVs secreted by the ECs under cellular stress [17]. Another study showed that cardiomyocyte (CM)-derived EVs under a diabetic condition cause an impairment of myocardial angiogenesis [18]. Moreover, Yeo et al. demonstrated that plasma and atrial exosome levels are increased in prolonged tachy-paced canine models [19]. Collectively, these observations raise the possibility that AF or AF-like tachyarrhythmic conditions enhance EV release from atrial tissue and that such EVs may affect other cell types, including vascular ECs.

Therefore, we hypothesized that the reduced eNOS expression and vascular endothelial dysfunction observed in AF may be mediated by EVs, and we investigated the effects of EVs derived from human plasma and from cultured atrial cardiomyocytes on vascular ECs in vitro.

## 2. Materials and Methods

### 2.1. Assessment of Endothelial Function

Endothelial function was assessed by an Endo-PAT 2000 device (Zoll Itamar Ltd., Caesarea, Israel) and estimated using the RHI. The RHI was measured in 12 patients with a sinus rhythm (SR), 10 patients with premature ventricular contractions (PVCs), and 22 patients with AF. Ethical approval of this study was given by the Institute of Science Tokyo’s Ethics Committee (approval number: M2018-140). Participants were recruited from outpatients attending the cardiology clinic at the Institute of Science Tokyo Hospital and were all without structural heart disease or other clinically apparent non-cardiac comorbidities. In this study, informed consent was signed by all the study participants.

### 2.2. Isolation of EVs from Human Plasma

Peripheral blood samples were obtained from the 3 groups (SR, *n* = 6; PVC, *n* = 5; AF, *n* = 6). Blood samples were collected in ethylenediaminetetraacetic acid-coated tubes and centrifuged at 1500× *g* for 10 min to separate the plasma. The plasma was then aliquoted to 500 μL each in 1.5 mL tubes.

EVs were isolated from 500 μL of plasma samples using the Total Exosome Isolation Kit (from plasma) (Thermo Fisher Scientific, Waltham, MA, USA), according to the manufacturer’s instructions. The EV pellet was resuspended in 250 μL of phosphate-buffered saline (PBS). To minimize protein contamination, proteinase K treatment was performed as recommended by the manufacturer. Accordingly, the final EV suspension corresponded to a two-fold concentration relative to the original plasma (i.e., 1 μL of EV suspension was equivalent to EVs derived from 2 μL of plasma).

### 2.3. Culturing Cardiomyocytes

HL-1 cells (murine atrial CMs) were kindly provided by Dr. Claycomb (University of Louisiana [20]). CMs were cultured with Claycomb culture medium (Sigma-Aldrich, St. Louis, MO, USA) supplemented with 10% fetal bovine serum (FBS), 1% penicillin/streptomycin, 2 mM of L-glutamine and 0.1 mM of norepinephrine on gelatin/fibronectin-coated plates in a humidified incubator at 37 °C and 5% CO_2_.

### 2.4. Electrical Stimulation of CMs and Isolation of EVs from Culture Media

CMs were seeded at 1 × 10^6^ cells per well in 6-well plates. After incubating for 24 h, the medium was exchanged with Dulbecco’s modified Eagle’s medium (DMEM) (Sigma-Aldrich, USA) containing 10% exosome-free FBS (Thermo Fisher Scientific, USA). CMs were paced with either regularly at 1 Hz or 10 Hz (1 Hz Reg-Paced, 10 Hz Reg-Paced) or irregularly at a mean frequency of 1 Hz or 10 Hz with 50% variability (1 Hz Irreg-Paced, 10 Hz Irreg-Paced). All four pacing models were applied for 24 h. Cells without electrical stimulation were kept as non-paced controls (Non-Paced). All cells were kept at 37 °C in a 5% CO_2_ humidified incubator.

After 24 h of pacing, 6 mL of the medium was collected, and EVs were isolated using Total Exosome Isolation Reagent (from cell culture media) (Invitrogen, Waltham, MA, USA) according to the manufacturer’s protocol. The EV pellet was resuspended in 180 μL PBS.

### 2.5. Culturing ECs

To culture ECs, we used the immortalized human umbilical vein cell line, EA.hy926 cells. ECs were cultured in DMEM with 10% FBS, 1% penicillin/streptomycin and 1% sodium pyruvate. ECs were seeded at 2.5 × 10^4^ cells/well in 96-well plates and incubated in a humidified incubator at 37 °C and 5% CO_2_.

### 2.6. Quantitative Evaluation of Isolated EVs

The particle size distribution and concentration of EVs from plasma and conditioned media of cultured CMs were evaluated using a SALD7500nano particle size analyzer (Shimadzu, Kyoto, Japan) and Videodrop interferometric light microscopy (Myriade, Paris, France).

The protein concentrations of isolated plasma-derived EVs and CM-derived EVs were measured using the Pierce BCA protein assay kit (Thermo Fisher Scientific, Waltham, MA, USA) according to the manufacturer’s protocol.

### 2.7. Labeling of Isolated EVs and Fluorescent Imaging of Labeled EVs

The isolated CM-derived EVs were labeled with a PKH26 Red Fluorescent Cell Linker Kit (Sigma-Aldrich, USA). EVs were stained according to the manufacturer’s protocol and centrifuged with an Amicon Ultra 15 centrifugal Filter Unit (Sigma-Aldrich, USA) at 4000× *g* for 30 min, then washed with PBS three times. Labeled EV concentrates were applied to cultured EA.hy926 cells, and after 12 h of incubation, the cells were washed with PBS and observed under a BZ-X710 fluorescence microscope (Keyence, Osaka, Japan).

### 2.8. Application of Isolated EVs to ECs

We applied 10 μL of plasma EVs or 18 μL of CM-derived EVs directly to the cultured ECs. After 24 h of EV treatment, cells were lysed and cDNA synthesized using a SuperPrepII cell lysis & RT kit (TOYOBO, Osaka, Japan). The cDNA synthesized were used to perform quantitative real-time PCR (qPCR) using Power SYBR Green Master Mix with a StepOnePlus Real-time PCR System (Applied Biosystems, Waltham, MA, USA). The data were analyzed using the ΔΔCT method, and *GAPDH* was used as an internal control. The primer sequences are listed in Table 1.

### 2.9. Transmission Electron Microscopy

Transmission electron microscopy (TEM) was performed to verify the morphology of the isolated EVs. Briefly, freshly isolated EV samples were absorbed onto carbon-coated copper grids for 10–20 min. The grids were then fixed with 1% glutaraldehyde for 5 min, followed by washing with distilled water. After air-drying, the grids were imaged using a JEM-1400 Flash transmission electron microscope (JEOL, Tokyo, Japan), operated at an accelerating voltage of 50 kV.

### 2.10. Western Blot Analysis

Western blotting was performed using the plasma-derived EV suspension and the corresponding plasma input at an equivalent plasma volume (1 μL EV suspension = EVs derived from 2 μL plasma). Samples were lysed in RIPA buffer containing protease inhibitors, separated by SDS-PAGE, and transferred onto polyvinylidene difluoride membranes. Membranes were blocked with 5% non-fat dry milk in TBS-T and incubated overnight at 4 °C with primary antibodies: mouse monoclonal anti-CD63 (sc-5275, Santa Cruz Biotechnology, Dallas, TX, USA, 1:1000), and goat polyclonal anti-ApoA1 (A81-104A, Bethyl Lab, Montgomery, TX, USA, 1:1000). After washing with TBS-T, membranes were incubated with HRP-conjugated secondary antibody for 1 h at room temperature. Protein signals were visualized using Chemi-Lumi One Ultra reagents (Nacalai Tesque, Kyoto, Japan). Chemiluminescent signals were detected and imaged using a Thermo iBright imaging system (Thermo Fisher Scientific, USA).

For EVs isolated from CM culture supernatants, Western blotting was performed using equal amounts of total protein from the EV preparations and the corresponding HL-1 cell lysates. Membranes were probed with the following primary antibodies: hamster monoclonal anti-CD81 (sc-18877, Santa Cruz Biotechnology, USA, 1:1000), and anti-GRP78 BiP (ab21685, Abcam Ltd., Cambridge, UK, 1:1000).

### 2.11. Statistical Analysis

All values were presented as the mean ± standard error of the mean (SEM), except for clinical measurements, which were presented as the mean ± standard deviation (SD). For comparisons among the three groups, continuous variables were analyzed using one-way analysis of variance (ANOVA), followed by Tukey’s post hoc test for multiple comparisons. Categorical variables were compared using Fisher’s exact test. A *p* value of less than 0.05 was considered statistically significant, and the data were analyzed statistically using Prism version 10.6.0 (GraphPad Software, San Diego, CA, USA).

## 3. Results

### 3.1. Endothelial Function Was Impaired in AF Patients

**Figure 1:**
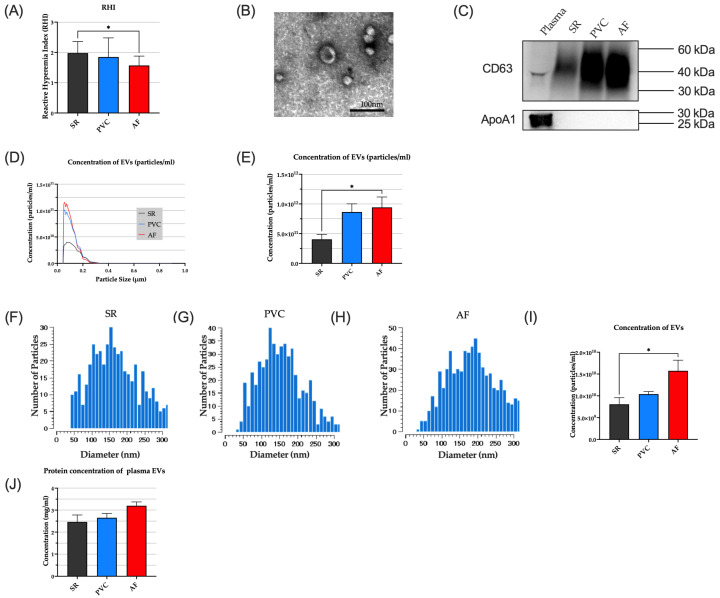
RHI and blood EV levels in AF. (**A**) RHI in the SR (*n* = 12), PVC (*n* = 10), and AF group (*n* = 22). (**B**) Representative TEM image of extracted EVs from human plasma. Scale bar: 100 nm. (**C**) Western blot analysis of extracted EVs for CD63 (**top**) and ApoA1 (**bottom**). (**D**) Particle size distribution of plasma EVs determined by nanoparticle analyzer. *n* = 6 each. (**E**) Particle concentration of plasma EVs determined by nanoparticle analyzer (SR, *n* = 6; PVC, *n* = 5; AF, *n* = 6). (**F**–**H**) Representative graphs showing particle distribution of plasma EVs determined by Videodrop analysis. (**I**) Particle concentration of plasma EVs determined by Videodrop analysis. *n* = 5 each. (**J**) Protein concentration of plasma EVs. *n* = 5 each. For quantitative analysis, multiple comparison was performed using one-way ANOVA with Tuckey’s post hoc test. *, *p* < 0.05. Error bars, SEM. AF, atrial fibrillation; EVs, extracellular vesicles; SR, sinus rhythm; PVC, premature ventricular contraction; RHI, reactive hyperemia index; TEM, transmission electron microscopy.

To assess endothelial dysfunction in AF, we measured the RHI in patients with AF. We also assessed the patients with PVCs, which present an irregular pulse. The patients’ characteristics in this study are described in Table 2. There were no statistically significant differences among the three groups in sex, age, body mass index (BMI), current smoking, or the prevalence of hypertension (HT) or diabetes mellitus (DM). It was found that the RHI in patients with AF was significantly decreased compared to that of the patients with SR (Figure 1A). In contrast, no significant reduction in RHI was observed in patients with PVCs compared with those in SR (RHI: SR, 1.98 ± 0.38; PVC, 1.85 ± 0.63; AF, 1.57 ± 0.31, SR vs. AF *p* = 0.049). Within the AF group, 41% of patients (9/22) had paroxysmal AF, and the remainder had persistent AF. Out of the 9 patients with paroxysmal AF, 7 patients (78%) were in AF during the RHI measurement. The RHI did not differ significantly between patients with paroxysmal and persistent AF (paroxysmal vs. persistent: 1.58 ± 0.29 vs. 1.57 ± 0.34, *p* = 0.93). These findings suggest that endothelial dysfunction in AF cannot be explained solely by the direct effects of irregular hemodynamic flow on the vascular endothelium and that additional pathophysiological mechanisms are implicated.

### 3.2. Plasma EV Level Was Elevated in Patients with AF

Plasma samples were collected from patients with SR, PVCs, or AF. After isolating the EVs from plasma, we identified the isolated EVs by TEM imaging, nanoparticle analysis and Western blotting. TEM imagery confirmed the presence of the EVs (Figure 1B). Western blot analysis confirmed the successful isolation and purity of EVs, showing the EVs-specific protein marker (CD63) and absence of non-EV contaminants (ApoA1) (Figure 1C). Compared with the SR group, the concentration of plasma-derived EVs isolated from patients with AF was significantly higher, whereas no significant increase was observed in patients with PVCs (Figure 1D–I). These results were consistent with the RHI-based evidence of endothelial dysfunction. In contrast, the total protein contents of the EV preparations did not differ significantly among the three groups (Figure 1J).

### 3.3. mRNA Expression of NOS3 Was Downregulated in ECs Treated with AF Patients’ Plasma EVs

**Figure 2:**
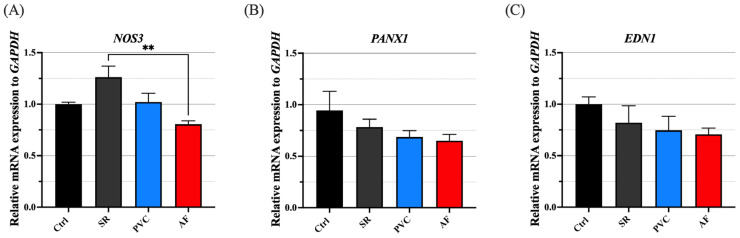
Gene expression of *NOS3*, *PANX1* and *EDN1* in plasma EV-treated ECs. (**A**–**C**) Relative gene expression of *NOS3*, *PANX1*, and *EDN1* in ECs treated with plasma EVs of patients with SR, AF and PVC. Ctrl indicates the ECs not treated with EVs. *n* = 5 each. For quantitative analysis, multiple comparison was performed using one-way ANOVA with Tuckey’s post hoc test. **, *p* < 0.01. Error bars, SEM. AF, atrial fibrillation; Ctrl, control; ECs, endothelial cells; EVs, extracellular vesicles; SR, sinus rhythm; PVC, premature ventricular contraction.

To evaluate whether circulating EVs contribute to endothelial dysfunction in AF, we exposed ECs to plasma-derived EVs in vitro and assessed *NOS3* mRNA expression by qPCR. In addition to *NOS3*, we examined *PANX1* and *EDN1*, genes whose expression changes have been implicated in impaired endothelial function [21,22,23]. *NOS3* mRNA expression was significantly reduced in ECs treated with plasma EVs from patients with AF compared with ECs treated with plasma EVs from patients with SR (Figure 2A). However, the mRNA expression levels of *PANX1* and *EDN1* showed no significant difference (Figure 2B,C). These findings suggest that, in AF, plasma EVs may contribute to endothelial dysfunction by downregulating *NOS3* expression.

### 3.4. Concentration of EVs Was Increased in Tachy-Paced Models

To confirm the effect of cardiac-derived EVs on ECs, we built in vitro tachy-paced models with mouse atrial-derived CMs, HL-1 cells. We established four pacing conditions: two tachypacing protocols (10 Hz Reg-Paced and 10 Hz Irreg-Paced) and two steady-state protocols (1 Hz Reg-Paced and 1 Hz Irreg-Paced).

**Figure 3:**
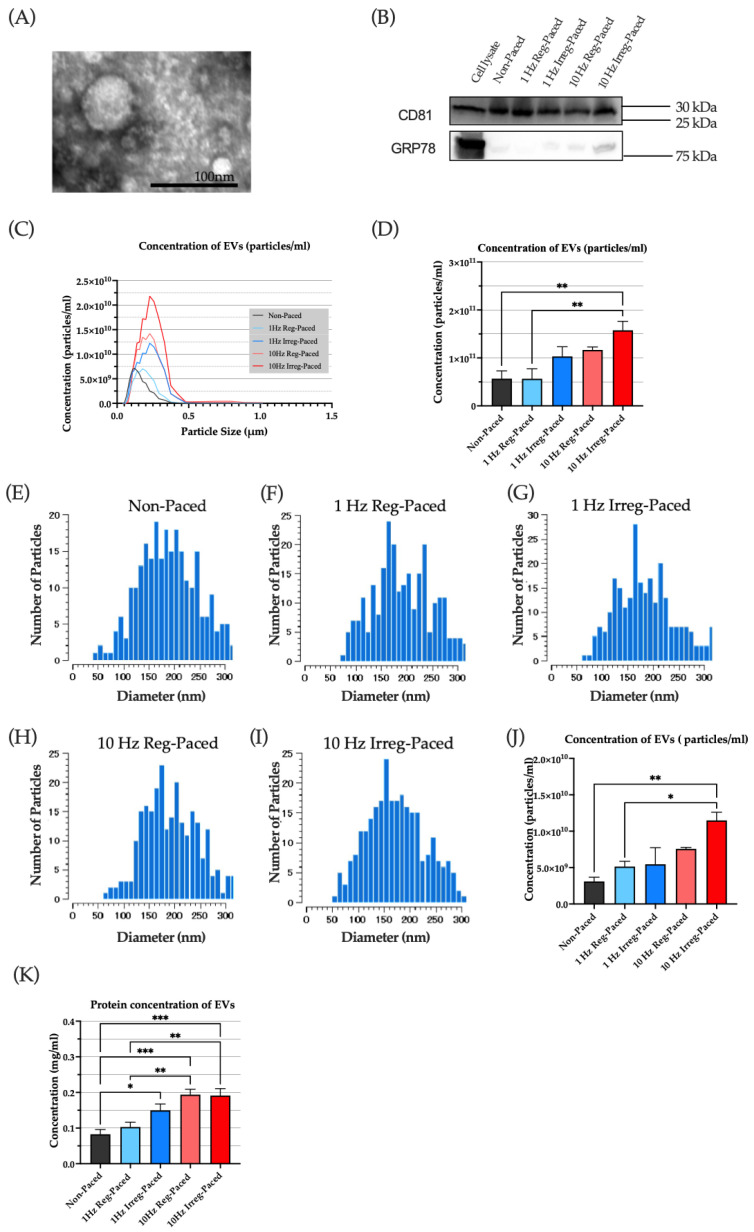
Identification of isolated cardiomyocyte-derived EVs. (**A**) Representative TEM image of EVs derived from paced HL-1 cardiomyocytes. Scale bar: 100 nm. (**B**) Western blot analysis of cardiomyocyte-derived EVs for CD81 (**top**) and GRP78 (**bottom**). (**C**) Particle size distribution of EVs released from HL-1 cardiomyocytes under Non-Paced or 1 Hz/10 Hz (Reg or Irreg) pacing. *n* = 6 each. (**D**) Particle concentration of cardiomyocyte-derived EVs determined by nanoparticle analyzer. *n* = 6 each. (**E**–**I**) Representative graphs showing particle distribution of EVs secreted by cardiomyocytes under various forms of electrical stimulation, determined by Videodrop analysis. (**J**) Particle concentration of EVs derived from cardiomyocytes, determined by Videodrop analysis. *n* = 3 each. (**K**) Protein concentration of cardiomyocyte-derived EVs. *n* = 6 each. For quantitative analysis, multiple comparison was performed using one-way ANOVA with Tuckey’s post hoc test. *, *p* < 0.05. **, *p* < 0.01. ***, *p* < 0.001. Error bars, SEM. EVs, extracellular vesicles; Irreg-Paced, irregular pacing; Non-Paced, non-pacing; Reg-Paced, regular pacing; TEM, transmission electron microscopy.

Following 24 h pacing of cultured CMs, EVs were isolated from the culture media and verified by TEM and western blotting. TEM imagery confirmed the presence of the EVs (Figure 3A). Western blotting analysis confirmed the presence of an EV-specific marker (CD81) and absence of a non-EV marker (GRP78), indicating the high purity of the isolated EVs (Figure 3B). The EV particle concentration was first assessed using a nanoparticle analyzer. As shown in Figure 3C,D, the 10 Hz Irreg-Paced group exhibited a significantly higher EV concentration than the non-paced group. Similarly, Videodrop analysis also showed a significant increase in particle concentration in the 10 Hz Irreg-Paced group compared to the Non-paced and 1 Hz Reg-Paced groups (Figure 3E–J). In contrast, the other pacing groups did not exhibit significant changes in EV concentration or particle size. The EV protein concentration was then measured. As shown in Figure 3K, all paced groups except the 1 Hz Reg-Paced group exhibited a significant increase in protein concentration, with both 10 Hz groups showing the highest levels.

Taken together, irregular rapid electrical stimulation of CMs appears to promote EV secretion.

### 3.5. Cellular Uptake of CM-Derived EVs by ECs

**Figure 4:**
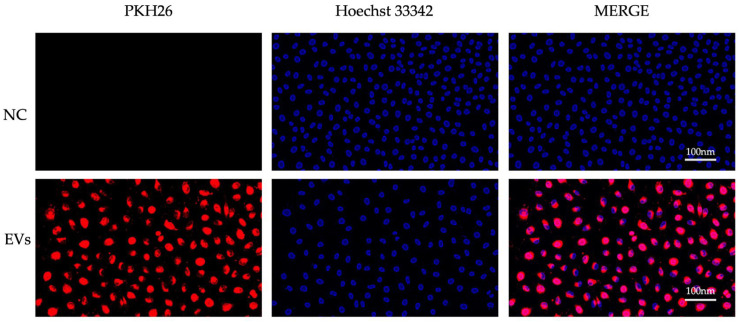
Cellular uptake of PKH26-labeled EVs by ECs. Fluorescence images of ECs treated with PKH26-labeled EVs. **Top row**: ECs exposed to non-labeled EVs (NC). Scale bar: 100 nm. **Bottom row**: ECs exposed to PKH26-labeled EVs. **Left column**: PKH26 (EV) signal (Red); **middle**: Hoechst nuclear stain (Blue); **right**: merged image. ECs, endothelial cells; EVs, extracellular vesicles; NC, negative control.

Next, to confirm the endothelial uptake of CM-derived EVs, isolated CM-derived EVs were labeled with PKH26 red fluorescent dye and incubated with ECs for 12 h. As shown in Figure 4, CM-derived EVs were internalized by ECs, despite the difference between the secreting and recipient cell types.

### 3.6. mRNA Expression of NOS3 Was Downregulated in ECs Treated with Tachy-Paced EVs

**Figure 5:**
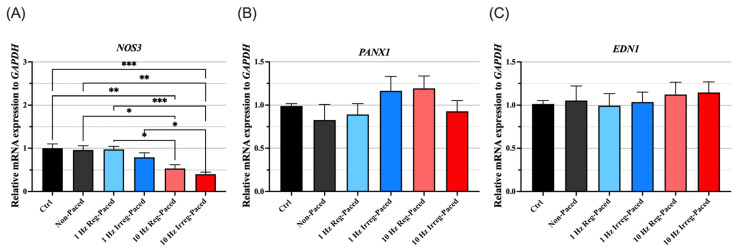
Gene expression of *NOS3*, *PANX1* and *EDN1* in CM-derived EV-treated ECs. (**A**–**C**) Relative gene expression of *NOS3*, *PANX1*, and *EDN1* in ECs treated with EVs derived from HL-1 cardiomyocytes under Non-Paced or 1 Hz/10 Hz (Reg or Irreg) pacing. Ctrl indicates the ECs not treated with EVs. *n* = 6 each. For quantitative analysis, multiple comparison was performed using one-way ANOVA with Tuckey’s post hoc test. *, *p* < 0.05. **, *p* < 0.01. ***, *p* < 0.001. Error bars, SEM. Ctrl, control; ECs, endothelial cells; EVs extracellular vesicles; Irreg-Paced, irregular pacing; Non-Paced, non-pacing; Reg-Paced, regular pacing.

Similarly to the experiments using human plasma-derived EVs, we evaluated the effects of cultured CM-derived EVs on ECs. EVs derived from CMs were applied directly to cultured ECs and incubated, after which mRNA expression was evaluated 24 h later. As shown in Figure 5, *NOS3* mRNA expression was significantly lower in ECs treated with CM-derived EVs from 10 Hz Reg-paced CMs and 10 Hz Irreg-Paced CMs than in Non-Paced EV-treated cells, with a tendency toward a greater reduction in the irregularly paced group. In contrast, *PANX1* and *EDN1* showed no significant differences across groups. These findings suggest that CM-derived EVs may impair endothelial function by suppressing *NOS3* at the transcriptional level, consistent with the results obtained using human plasma-derived EVs.

## 4. Discussion

This study yielded several key findings. First, patients with AF exhibited endothelial dysfunction, as indicated by a lower RHI, together with higher levels of circulating EVs, whereas these changes were not observed in patients with SR or PVCs. Second, plasma-derived EVs from patients with AF suppressed *NOS3* expression in ECs in vitro. Third, atrial CMs subjected to irregular rapid pacing in vitro released greater amounts of EVs, and these CM-derived EVs similarly reduced endothelial *NOS3* expression. Taken together, these data suggest that the irregular, rapid atrial activation in AF may enhance EV release, which could be linked to endothelial dysfunction in AF. Notably, a key novel finding of our study is that EVs from patients with AF and from irregularly rapid-paced CMs suppress *NOS3* gene expression in ECs.

Endothelial dysfunction in AF is well recognized. However, vascular endothelial function—particularly as assessed by the RHI—is also influenced by multiple factors beyond AF, including age, sex, BMI, smoking status, and various comorbidities such as HT and DM [24,25]. In the present study, we evaluated patients with AF as well as those with PVCs and individuals in SR. Baseline characteristics did not differ significantly among the groups, and our findings therefore suggest an association between AF and a lower RHI. Moreover, because vascular endothelial dysfunction has been reported to improve after restoration of SR, AF itself is considered to contribute to endothelial dysfunction [10,11,12,13]. Consistent with these observations, our data also support the notion that AF may underlie the reduced RHI observed in our AF cohort.

A leading hypothesis for AF-related endothelial dysfunction is altered shear stress secondary to pulse irregularity [14]. However, the precise mechanisms have not been fully elucidated. In the present study, we hypothesized that factors beyond hemodynamics contribute to endothelial dysfunction in AF, with a particular focus on EVs. We found that EVs derived from patients with AF suppressed *NOS3* expression in ECs in vitro. Given the established role of *NOS3*/eNOS in maintaining NO bioavailability and endothelial function, these results suggest a plausible mechanism by which AF-derived EVs may be linked to endothelial impairment and the adverse vascular phenotype observed in AF. Nevertheless, whether this EV-mediated downregulation of *NOS3* occurs in vivo and contributes causally to endothelial dysfunction requires further investigation.

Consistent with previous studies, we observed an elevated burden of circulating EVs in patients with AF [26]. However, the biological consequences of EVs in AF may reflect not merely a quantitative increase but also qualitative alterations in cargo composition. In the present study, we did not characterize EV cargo or delineate the molecular mechanism by which EVs downregulated *NOS3* expression; therefore, further studies are warranted to identify the responsible EV components and clarify the underlying pathways.

A previous report indicated that the proportion of CM-derived EVs within the circulating EV pool is low [27], raising uncertainty as to whether the increase in circulating EVs observed in AF can be explained solely by augmented release from CMs, particularly atrial CMs. Accordingly, whether CM-derived EVs, particularly those from atrial CMs, account for the *NOS3* suppression observed with patient plasma-derived EVs remains unclear. Potential contributions from EVs originating from non-cardiomyocyte cell types could be considered and warrant further exploration.

A key strength of this study is that we included not only SR controls but also patients with PVCs as a disease-control group, allowing us to distinguish AF-associated endothelial dysfunction from the effects of rhythm irregularity alone. In addition, complementary in vitro experiments corroborated the functional effects of patient-derived EVs observed in the clinical cohort.

Our study also has some limitations. First, the sample size was relatively small. Further studies in larger cohorts are needed. Second, our mechanistic assessment evaluated only three endothelial-related targets. Future studies should incorporate not only comprehensive profiling of EV cargo but also paced-CM transcriptomic analysis and molecular profiling and functional assays in ECs to better link gene expression changes to endothelial physiology. Third, our in vitro experiments included EVs derived from different sources, including human plasma and mouse CMs in culture, and thus involve a cross-species approach. Because many potential EV cargos, including proteins and miRNAs, are highly conserved between mice and humans, cross-species EV experiments are not ideal but can be acceptable in EV research; however, the results require careful interpretation. Importantly, we also confirmed a consistent effect in a human-to-human setting in the present study, which supports our overall interpretation despite the cross-species component. Nevertheless, future studies using EVs released from human CMs under irregular rapid pacing conditions (e.g., iPSC-derived cardiomyocytes) will be needed to further validate these findings.

In conclusion, circulating EVs in AF can be associated with reduced *NOS3* expression in ECs. Our findings provide new insight into the pathological role of EVs in AF.

## Figures and Tables

**Table 1:** Primer sequences for qPCR.

Gene Name (Species)	Forward Primer	Reverse Primer
*GAPDH* (*Homo sapiens*)	TCGGAGTCAACGGATTTGG	GGCAACAATATCCACTTTACCAGAGT
*NOS3* (*Homo sapiens*)	CTCGTCCCTGTGGAAAGACAA	ACGATGGTGACTTTGGCTAGCT
*PANX1* (*Homo sapiens*)	AAAGAGTGCGGTGACCTTG	GGGTACTTGAAGTGGCTTTCAG
*EDN1* (*Homo sapiens*)	TGGGAAAAAGTGTATTTATCAGCA	TTTGACGCTGTTTCTCATGG

**Table 2:** The patients’ characteristics.

	SR (*n* = 12)	PVC (*n* = 10)	AF (*n* = 22)
Male sex	7 (58%)	5 (50%)	15 (68%)
Age	67.1 ± 10.9	55.0 ± 13.7	67.4 ± 15.0
BMI	23.3 ± 0.81	23.3 ± 3.6	24.7 ± 4.5
Current smoker	0 (0%)	1 (10%)	3 (14%)
HT	0 (0%)	2 (20%)	1 (5%)
DM	0 (0%)	1 (10%)	3 (14%)

Categorical variables are presented as *n* (%), and continuous variables are presented as mean ± standard deviation. SR, sinus rhythm; PVC, premature ventricular contraction; AF, atrial fibrillation; BMI, body mass index; HT, hypertension; DM, diabetes mellitus.

## Data Availability

The original contributions presented in this study are included in the article/Supplementary Material. Further inquiries can be directed to the corresponding author.

## Supplementary Materials

The following supporting information can be downloaded at: https://www.mdpi.com/article/10.3390/jcm15041399/s1, Figure S1: Western Blot Membrane Images.

## Abbreviations

The following abbreviations are used in this manuscript:

SR	Sinus rhythm
AF	Atrial fibrillation
EV	Extracellular vesicle
PVC	Premature ventricular contraction
RHI	Reactive hyperemia index
EC	Endothelial cell
CM	Cardiomyocyte
Non-Paced	Endothelial cell model without electrical stimulation
Reg-Paced	Regularly paced endothelial cell model
Irreg-Paced	Irregularly paced endothelial cell model
