# Supplementary material for: Circulating Extracellular Vesicles Downregulate NOS3 Expression in Endothelial Cells in Atrial Fibrillation
Source: J Clin Med. 2026 Feb 10;15(4):1399. doi: 10.3390/jcm15041399 (PMC12942163; doi:10.3390/jcm15041399)

Full length images of blot for Fig 1. C (Upper). Cropped area is indicated in red square.

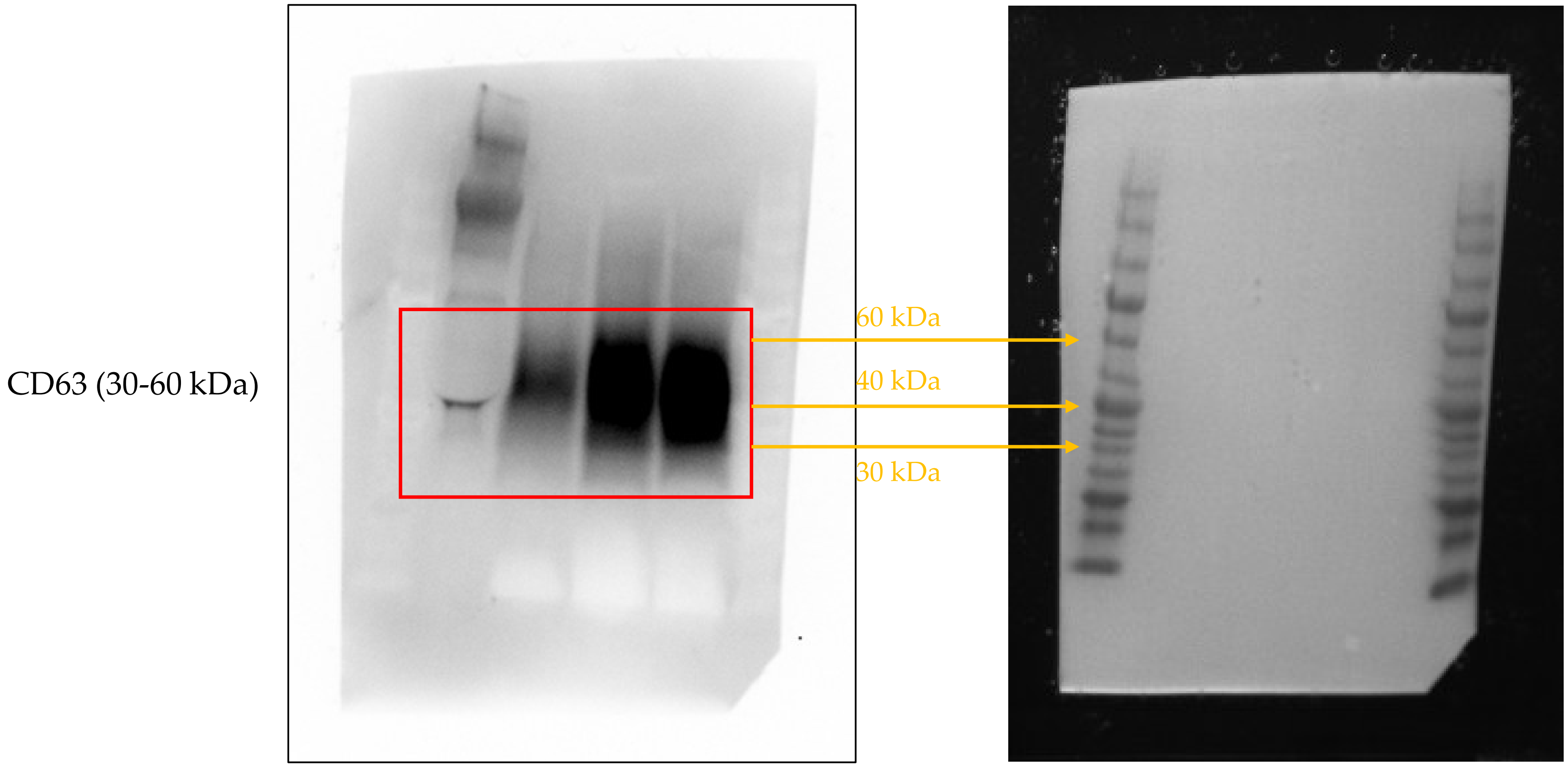

Full length images of blot for Fig 1. C (Lower). Cropped area is indicated in red square.

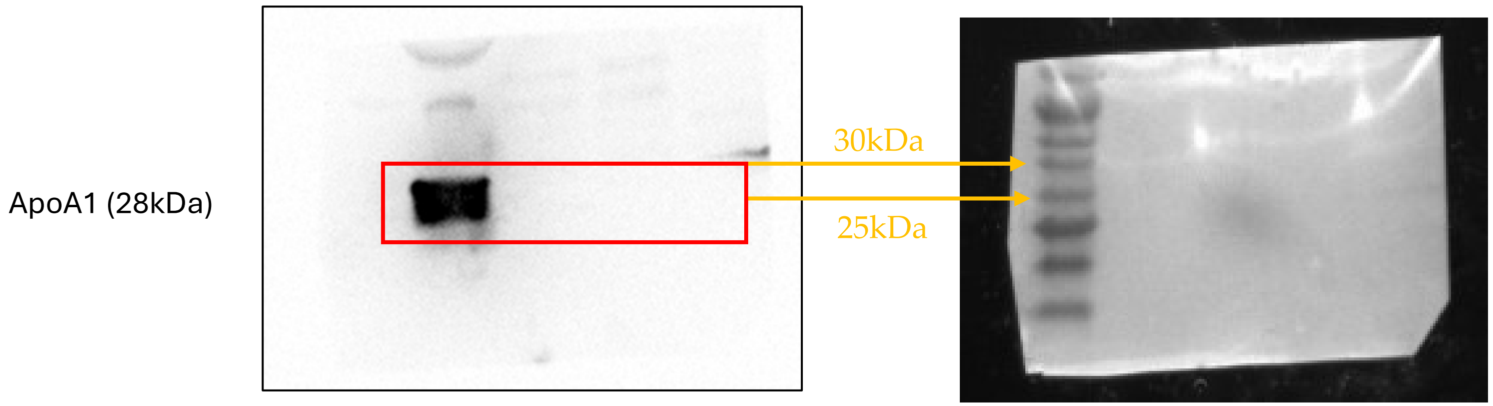

Full length images of blot for Fig 3. B (Upper). Cropped area is indicated in red square.

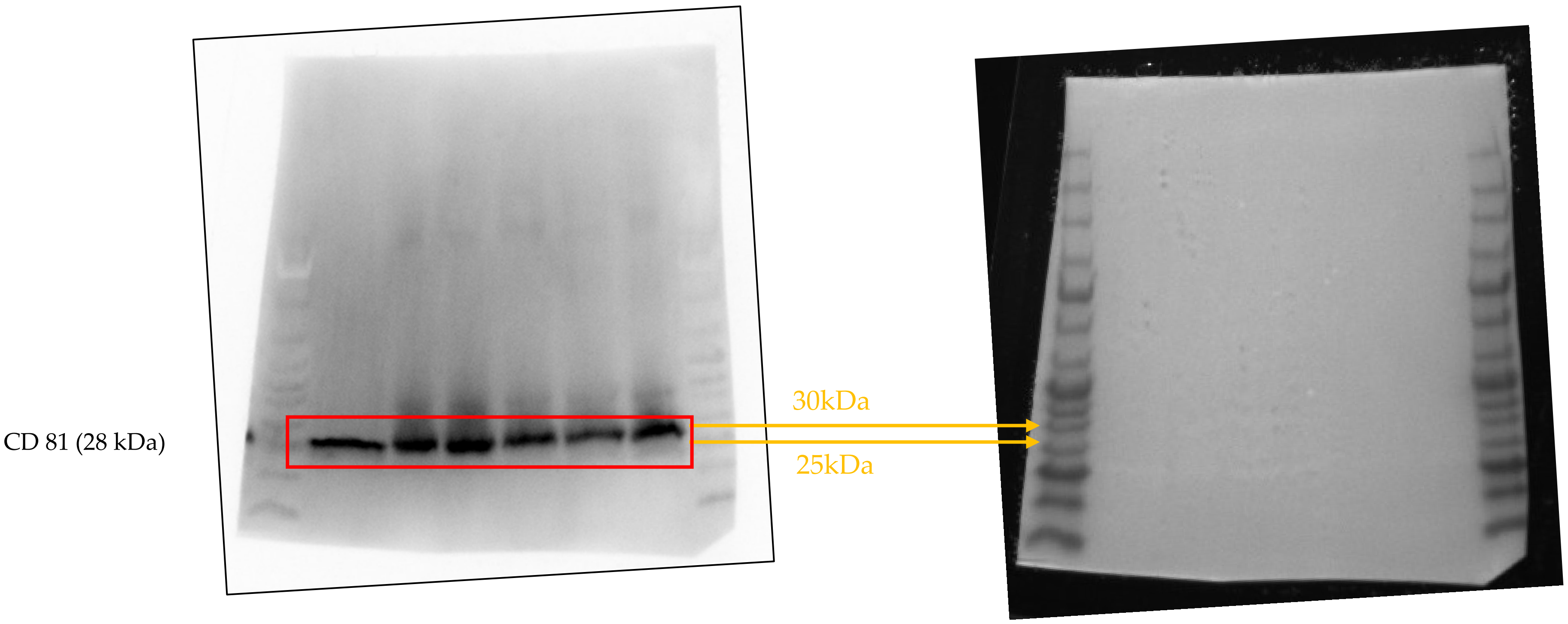

Full length images of blot for Fig 3. B (Lower). Cropped area is indicated in red square.

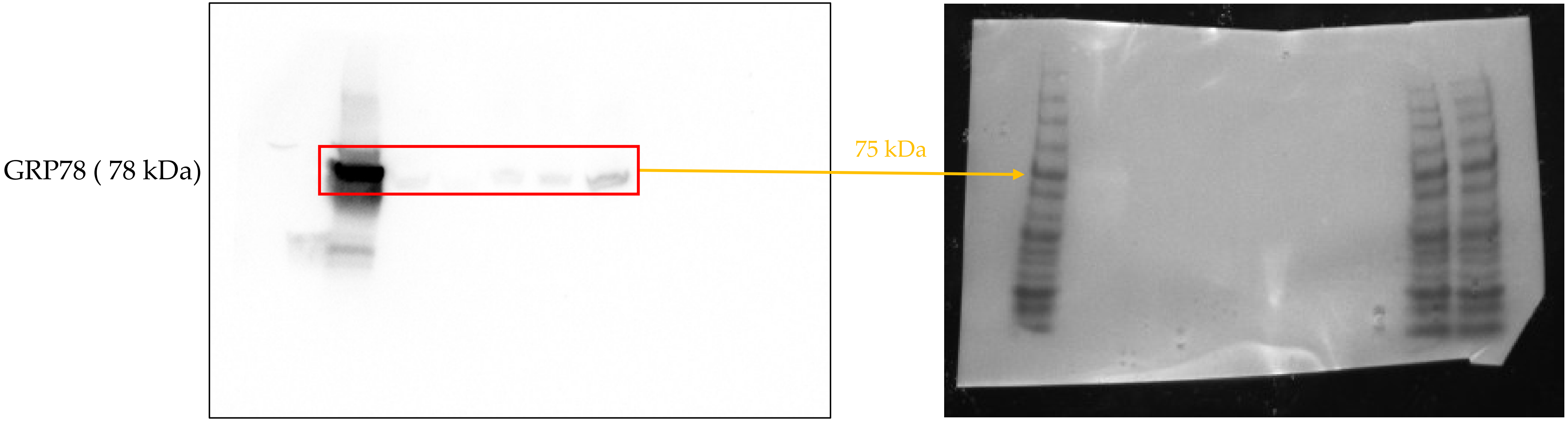

Supplement: Supplementary file 1 [file jcm-15-01399-s001.zip › jcm-4094457-supplementary.pdf]
